# An Intronic miRNA Regulates Expression of the Human Endothelial Nitric Oxide Synthase Gene and Proliferation of Endothelial Cells by a Mechanism Related to the Transcription Factor SP-1

**DOI:** 10.1371/journal.pone.0070658

**Published:** 2013-08-05

**Authors:** Limei Yan, Min Kang, Zhengqi Qin, Wenyu Zhang, Yumei Li, Hesheng Ou

**Affiliations:** 1 Center of Biochemistry Research, University of South China, Hengyang, Hunan, P. R. China; 2 The First Affiliated Hospital, Guangxi Medical University, Nanning, Guangxi, P. R. China; 3 College of Pharmacy, Guangxi Medical University, Nanning, Guangxi, P. R. China; University of British Columbia, Canada

## Abstract

**Objective:**

This study was to investigate the molecular mechanisms underlying the 27nt-miRNA-mediated regulation of expression of the endothelial nitric oxide synthase (eNOS) gene.

**Methods:**

Cell lines overexpressing 27nt-miRNA or its mutant were established by transfecting the miRNA expression vector into the endothelial cells. eNOS mRNA and protein expression were examined by RT-PCR and Western Blotting, respectively. Luciferase activity reporter system was used to study the target of 27nt-miRNA.

**Results:**

The results showed that overexpression of 27nt-miRNA significantly inhibited eNOS mRNA level and protein expression, and reduced the eNOS transcriptional efficiency. Such inhibitory effects of 27nt-miRNA were attenuated by the sequence mutations in 27nt-miRNA. Interestingly, the transcription factor SP-1 expression was reduced by 27nt-miRNA. Meanwhile, overxpression of SP-1 protein partially restored eNOS expression, and rescued the 27nt-miRNA-mediated reduction of endothelial cell proliferation. Moreover, certain sites in the SP-1 mRNA were found to be the direct target of 27nt-miRNA by a luciferase reporter system.

**Conclusions:**

These results demonstrate that the 27nt-miRNA suppresses eNOS gene expression and SP-1 expression in vascular endothelial cells. The 27nt-miRNA directly target to SP-1 mRNA, thereby contributing to proliferation of endothelial cells.

## Introduction

MicroRNAs (miRNAs) are a group of small non-coding RNAs identified in a variety of organisms [1], [2]. Most miRNAs are considered as intergenic, located in the noncoding regions between genes and transcribed by unidentified promoters. However, another group of noncoding RNAs were discovered in 2003, and described as intronic miRNAs from the intron regions of gene transcripts [3]. Initially, introns were thought to be a huge genetic waste in gene transcripts, since they occupied a large portion of noncoding sequences in the protein-coding DNA. Recently, the importance of intronic miRNAs has been recognized by the findings that intronic miRNAs are structurally and functionally similar to intergenic miRNAs [4]. Approximately 10–30% of the spliced introns are exported into cytoplasms, which show a moderate half-life in the vertebrate cells [5]. Intronic miRNAs are present in vertebrate cells, and critically involved in the regulation of host gene expression [6].

The human endothelial nitric oxide synthase (eNOS) gene (7q35–36), containing 26 exons and 25 introns, is expressed mainly in endothelial cells [7]. Several polymorphisms have been described in its genetic structure, including G894T SNP in exon 7, T786C in the promoter, 35 CA repeats in intron 13 and a 27-nucleotide (27nt) variable number of tandem repeats (VNTR) in intron 4 [7], [8]. Particularly, the 27nt VNTR has been recognized as a polymorphic genotype linked to cardiovascular diseases such as atherosclerosis and coronary heart disease, and type-2 diabetes [9]–[11]. It was found that the 27nt repeats in the eNOS intron 4 act as a cis-acting regulator of eNOS expression [12], [13].

We previously reported that the 27nt VNTR in intron 4 of eNOS is the origin of intronic miRNAs (termed as 27nt-miRNA) [14]. We found that this 27nt-miRNA could inhibit eNOS expression at the level of protein translation and mRNA transcription [14], [15]. Interestingly, we recently reported that overexpressed 27nt-miRNA significantly suppressed eNOS expression and endothelial cell proliferation via inhibition of STAT3 signaling [16], suggesting that some nuclear transcription factors (TFs) might be involved in regulation of gene expression by the miRNA.

Several TFs including Sp-1, Ap-1, Ets, and NF-1, have binding sites in the 5′-regulatory region of eNOS gene, and are responsible for accurate regulation of eNOS gene basal transcription through two *cis* elements at −104/−95 and −144/−115 positions [17]. Recent evidence indicates that miRNAs are critical to the expression of TFs [18] or directly target some TFs [19]. We have observed that the exogenously transfected 27nt miRNA could enter the nucleus of endothelial cells [14], and nuclear proteins could bind to the 27nt repeats to enhance eNOS gene expression [15]. It is intriguing to determine whether TFs directly interact with intronic 27nt miRNAs, and/or adapt to the intron genotype polymorphisms during gene transcription initiation and/or expression. In our previous study [14], three bases (8^th^–10^th^) in the 5′-terminal region of 27nt-miRNA were altered and the results indicated that the 5′-terminal region of 27nt-miRNA is important for its binding to the target genes. In the current study, another three bases (22^nd^–24^th^) in the 3′-terminal region of 27nt-miRNA were mutated to investigate if SP-1 mediates the function of 27nt-miRNA in proliferation of endothelial cells. We hypothesized that SP-1 plays an important role in the 27nt-miRNA-mediated down-regulation of eNOS expression, therefore contributing to regulation of vascular endothelial cell proliferation.

## Materials and Methods

### Construction of miRNA-expressing Plasmids

The sequence of 27nt repeat in intron 4 of eNOS gene and its double-length and mutants were used to design the hairpin miRNA, according to the method described previously [16]. To investigate the polymorphisms of the miRNAs, we designed a mutant oligonucleotide with 3 mutations at sites 13, 14, and 15 (Table 1), which located in the center of the 27nt sequence. A loop of 9 nucleotides (5′-UUCAAGACA-3′) was used for the production of miRNAs. We used 27nt sequence from the Luciferase gene as control. The plasmids contained a G418 antibiotic selection marker to allow identification of positively transfected cells, and subsequent establishment of stable cell lines (referred as stable cell lines No. 1, 2, and 3 in Table 1). The sequences and orientations of the inserts were verified by sequencing. The sequence in control vector had no similarity to the human genome. All the sequences and the names of plasmids were listed in Table 1.

**Table 1:** Sequences of oligonucleotides used in this study.

Name	Sequences	Cell lines
Control (Cont)	5′-GTCTGACAGTTACCAATGCTTAATCAG -3′	1
wt-27nt-miR	5′-GAAGTCTAGACCTGCTGCAGGGGTGAG -3′	2
mut-27nt-miR	5-GAAGTCTAGACCTGCTGCAGGCCAGAG -3′	3
eNOS_forward	5′-AGGAACCTGTGTGACCCTCA-3′	
eNOS_reverse	5′-CGAGGTGGTCCGGGTATCC-3′	
Sp-1_forward	5′-AGGTGCACCAGCTTCCAGGCCTG-3′	
Sp-1_reverse	5′-CCAGGTCCATGAAGGCCAAGTTG-3′	
β-actin _forward	5′-CTGGAACGGTGAAGGTGACA-3′	
β-actin _reverse	5′-AAGGGACTTCCTGTAACAATGCA-3′	

Note: The underlined letters indicated mutated sites.

### Cell Culture, Transfection and Establishment of Stable Cell Lines

Human aortic endothelial cells (HAECs) (Cell Application, Santiago, CA) were cultured in EBM-2 Bulletkit with 3% FBS. Primary cells between passage 4 and 10 were used in the experiments and in the establishment of stable cell lines. For transfection, cells were grown up to 90% confluence and transfected with siRNAs or miRNA plasmids with Lipofectamine 2000 (Invitrogen, Calsbad, CA, USA) by incubating with OptiMem-I media for 4 h. The cells were then transferred into fresh EBM-2 with 3% FBS. To establish the stable cell lines of miRNA overexpression, G418 (1 mg/ml; Gibco™ Invitrogen, Carlsbad, CA, USA) was added into the media 2 days after transfection with changes of selection medium once a week. The transfected cells expressing neomycin resistance gene could survive in the selection media with elimination of the non-transfected cells. The cells were individually picked 2 weeks later using conventional cloning techniques for expansion. Passages 2–10 of the stable cell lines were identified by verifying the miRNA expression with Northern blotting and *in situ* hybridization, and used for experiments.

### Real-Time RT-PCR

Total RNA was extracted from the cells with TRIzol reagent according to the manufacture’s protocol. After treatment with RNase-free DNase I to remove any genomic DNA contamination, the mRNAs were reverse-transcribed into cDNAs for real-time PCR analysis as described [15]. The primers were given in Table 1. All data were presented as mean ± SD of three separate experiments in duplicates.

### Nuclear Run-on Assay

Nuclear run-on assays were performed with the method as described ^(1)^. Briefly, nuclei were isolated from the cultured HAECs, and immediately stored at −80°C until the assay. For reaction of nuclear run-on transcription, 5×10^6^ nuclei were incubated in a reaction buffer (5 mM Tris·HCl, pH 8.0, 2.5 mM MgCl_2_, 150 mM KCl, 2.5 mM each of ATP, GTP, CTP) and biotin-16-UTP at 30°C for 45 min in a final volume of 60 µl. The reaction was terminated by adding 1,000 units of RNase-free DNase and incubating for 10 min at 37°C. The nuclei were then lysed by lysis buffer containing 10 mM Tris·HCl, 1% SDS, and 5 mM EDTA. Total RNA was then extracted with TRIzol reagent, precipitated with ethanol, and resuspended in 50 µl of RNase-free H_2_O. Biotinylated RNAs were purified and prepared for quantitative real-time RT-PCR analysis.

### Western Blotting

Cells were washed, harvested, and prepared for Western blotting as described [15]. A total of 30 µg of proteins were separated using 8% SDS-PAGE and transferred to a nitrocellulose membrane. The membrane was then incubated with the primary antibodies against eNOS (Cell Signaling Technology, Boston, MA,USA, 1∶1000), SP-1(Santa Cruz, CA, USA, 1∶1000), Ap-1 (ABZOOM, Dallas, TX, USA, 1∶1000), and actin (Cell Signaling Technology, Boston, MA,USA, 1∶5000) in the 5% non-fat milk in TBST at room temperature for 1 hr, followed by exposure to a goat anti-rabbit or anti-mouse secondary antibody (Cell Signaling Technology, Boston, MA,USA,) conjugated with horseradish peroxidase. Signals of the immunoreactive bands were visualized using the ECL detection system (Invitrogen, Calsbad, CA, USA).

### Statistical Analyses

All data were expressed as mean ± SEM of three separate experiments unless specified otherwise. Independent Student’s *t*-test was used for comparisons between groups. One-way ANOVA was applied for multiple group comparisons with post hoc Bonferroni correction for multiple comparisons using SPSS 12.0 for Windows (SPSS, Chicago, USA). The differences were considered statistically significant when *p*<0.05.

## Results

### Intronic 27nt miRNA Significantly Inhibits eNOS Expression in Endothelial Cells

**Figure 1:**
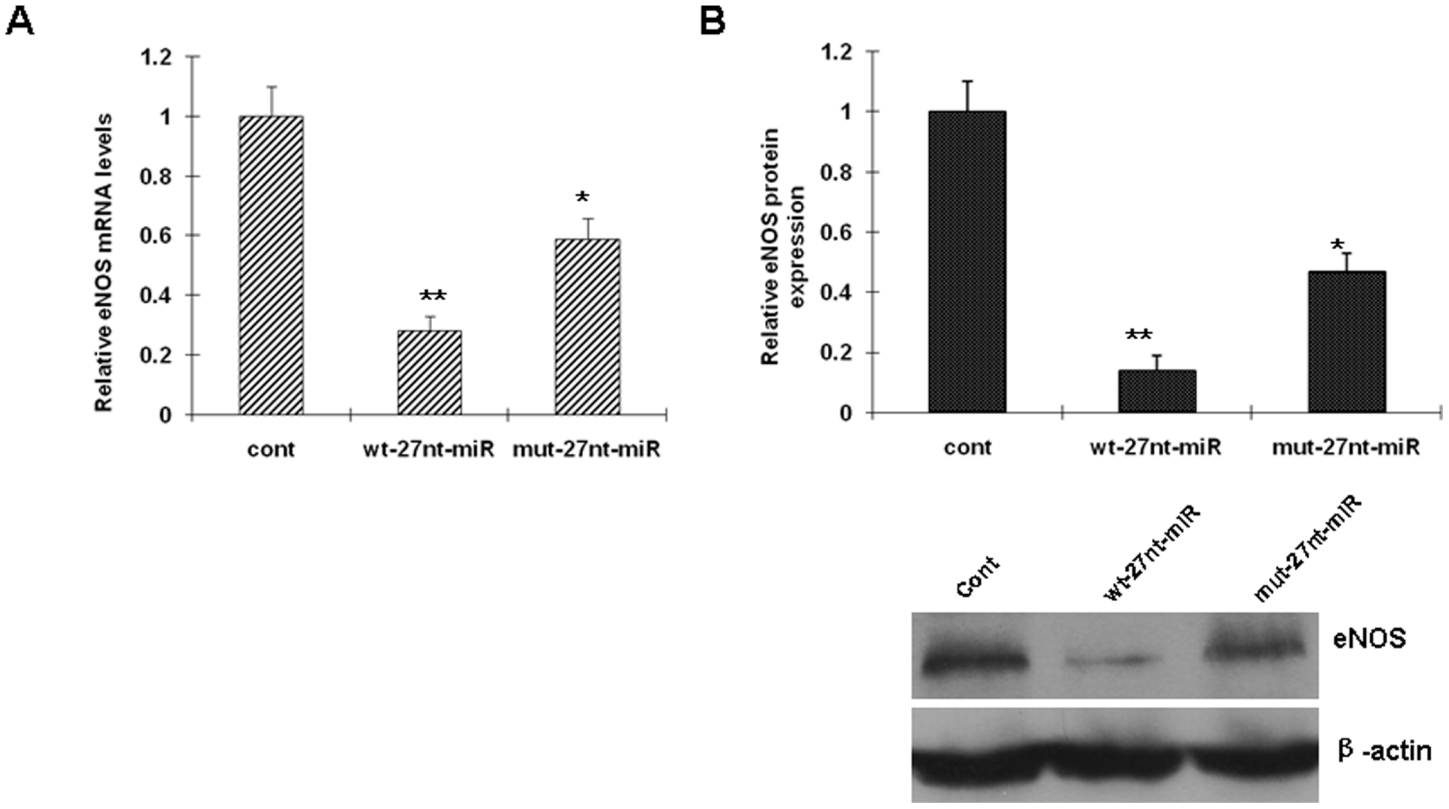
Intronic 27nt miRNA significantly inhibits eNOS expression. HAECs were transfected with different miRNA plasmids. Total RNAs and proteins were isolated from the cells and analyzed by RT-PCR (A) and Western blotting (B), respectively. cont, cells transfected with control plasmid; wt-27nt-miR, cells transfected with plasmid expressing wild-ytpe 27-nt-miRNA; mut-27nt-miR, cells transfected with plasmid expressing mutant 27-nt-miRNA.*, *P*<0.05, **, *P*<0.01 vs control by independent Student’s *t* test.

To test whether the 27nt miRNA influences eNOS expression at both the mRNA and protein levels, endothelial cells were transfected with the 27nt miRNA plasmid or the control for 24 hrs, then the RNA and protein were isolated and analyzed by RT-PCR and Western Blotting, respectively. As shown in Fig. 1A, eNOS mRNA levels (measured by RT-PCR) were significantly decreased by 72.0% (0.28±0.05 vs. 1.00±0.10, P<0.01) and 41.0% (0.59±0.07 vs 1.00±0.10, *P*<0.05) by wt-27nt-miRNA and mut-27nt-miRNA, respectively, when compared with control (cont). The extent of the eNOS mRNA decrease by wt-27nt miRNA was significantly greater than mut-27nt miRNA (0.28±0.05 vs. 0.59±0.07, *P*<0.01), suggesting that mutation of miRNA decreases its ability to inhibit eNOS expression.

**Figure 2:**
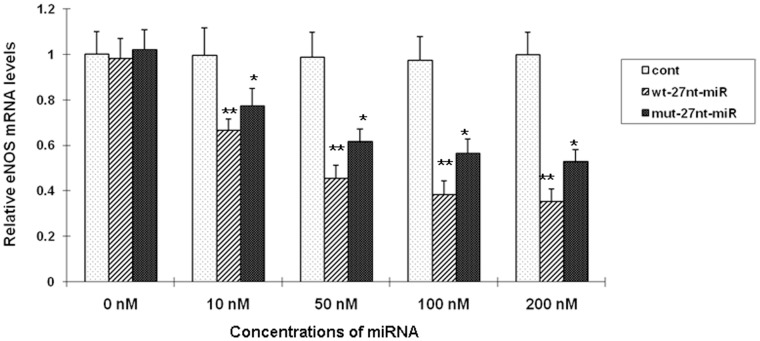
Effect of miRNAs on eNOS transcription efficiency *in vitro*. The nuclei of HAECs were isolated and prepared for the nuclear run-on transcription reaction to determine the eNOS transcription efficiency (the relative mRNA levels of eNOS and β-actin) in the presence of different miRNAs with RT-PCR. The concentrations of the miRNA oligonucleotides, wt-27nt-miRNA or mut-27nt-miRNA, are 0, 10, 50, 100, or 200 nM. Cont, cells transfected with control plasmid; wt-27nt-miR: cells transfected with plasmid expressing wild-ytpe 27-nt-miRNA; mut-27nt-miR: cells transfected with plasmid expressing mutant 27-nt-miRNA. *, *P*<0.05; **, *P*<0.01 vs control (n = 3).

Consistent with the changes in the mRNA levels, eNOS protein expression was decreased by 86.0% (0.14±0.05 vs. 1.00±0.10, *P*<0.01) and 53.0% (0.47±0.06 vs. 1.00±0.10, *P*<0.01) by wt-27nt-miRNA and mut-27nt-miRNA), compared with the control, respectively (Fig. 2B). The inhibitory effect of mut-27nt-miRNA on eNOS protein expression was significantly less than the wt-27nt-miRNA (0.47±0.06 vs. 0.14±0.05, *P*<0.01). These results suggest that the intronic 27nt miRNA significantly inhibits eNOS expression in endothelial cells.

### Effect of miRNAs on the Efficiency of eNOS Transcription in vitro

To evaluate the effect of variant miRNA duplexes on eNOS transcription efficiency *in vitro*, the nuclei of endothelia cells were isolated and the nuclear run-on assay was performed. Nuclei were isolated from HAECs without plasmid transfection, and used to establish the basal level of eNOS transcription efficiency that was measured as relative eNOS mRNA levels over β-actin. As shown in Fig. 2, the eNOS transcriptional efficiency was significantly decreased by wt-27nt miRNA or mut-27nt miRNA in a dose-dependent manner. When the concentrations of 27nt miRNA was increased to 10, 50, 100, and 200 nM, the eNOS mRNA levels were significantly decreased by 33.2% (0.665±0.051 vs 0.995±0.122, *P*<0.05), 54.0% (0.454±0.058 vs 0.986±0.110, *P*<0.01), 60.7% (0.382±0.062 vs 0.973±0.106, *P*<0.01), and 64.7% (0.352±0.056 vs 0.996±0.102, *P*<0.01) as compared with control, respectively. At the same concentrations, the mut-27nt miRNA also reduced eNOS mRNA levels by 22.5% (0.771±0.078 vs 0.995±0.122, *P*<0.05), 37.7% (0.614 vs 0.986±0.110 *P*<0.01), 42.2% (0.562±0.065 vs 0.973±0.106, *P*<0.01), and 47.1% (0. 527±0.054 vs 0.996±0.102, *P*<0.01) as compared with control, respectively. These results suggest that the mut-27nt miRNA was less potent in suppressing eNOS transcriptional efficiency than wt-27nt miRNA. The control, which is 27nt RNA complementary sequence to the coding sequence of luciferase gene, did not change the eNOS transcription efficiency as expected (Fig. 2).

### SP-1 Expression is Negatively Regulated by miRNA

**Figure 3:**
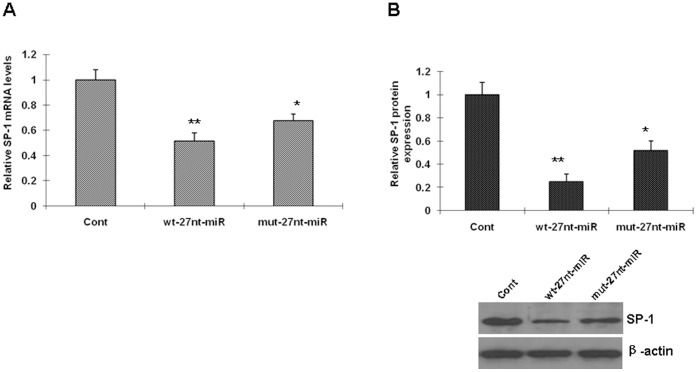
Sp-1 expression is negatively regulated by miRNA. The stable endotheial cell lines transfected with the wild-type 27-nt-miRNA, mutant 27-nt-miRNA, or the control plasmids were used for analysis of Sp-1 expression. Sp-1 mRNA and protein were measured by RT-PCR and Western Blotting, respectively. Cont, cells transfected with control plasmid; wt-27nt-miR: cells transfected with plasmid expressing wild-type 27-nt-miRNA; mut-27nt-miR: cells transfected with plasmid expressing mutant 27-nt-miRNA. *, *P*<0.05; **, *P*<0.01 vs control (n = 3).

Because the 27nt miRNA decreases the transcriptional efficacy of eNOS *in vitro* as determined by the nuclear run-on assay, we continued to test whether the miRNA affects the expression of nuclear transcription factor SP-1, which is a positive regulator in eNOS transcription. We analyzed the SP-1 mRNA level and protein expression in the cell lines transfected with the control plasmid, wt-27nt-miRNA, or mut-27nt-miRNA. As showed in Fig. 3 A, SP-1 mRNA levels were decreased in cells transfected with plasmids expressing wt-27nt miRNA by 48.7% (0.513±0.066 vs 1.000±0.079, *P*<0.01) and in cells transfected with plasmids expressing mut-27nt miRNA by 32.4% (0.676±0.051 vs 1.000±0.079, *P*<0.01) compared with control, respectively. We also analyzed the protein expression by Western Blotting. Compared with control, SP-1 protein expression was significantly decreased by 74.7% (0.253±0.063 vs 1.000±0.105) and 48.2% (0.518±0.082 vs 1.000±0.105) by wt-27nt miRNA and the mut-27nt miRNA, respectively (Fig. 3B). These results suggested that SP-1 mRNA level and protein expression were inhibited by 27nt miRNA.

### SP-1 may be a Direct Target of 27nt-miRNA

Considering that SP-1 mRNA level is reduced in the cell line transfected with the 27nt miRNA, we speculated that SP-1 mRNA is the target of 27nt miRNA. Through computational prediction, we found that sequence 5′-TCACCCC-3′at 7443–7449 bases of SP-1 mRNA (Genbank accession NM_138473.2) is matched to the sequence 5′-GGGGTGA-3′ within 27nt miRNA. In order to investigate if SP-1 is one target gene of the miRNA, we constructed luciferase-reporter plasmids that contain the wt (5′-TCACCCC-3′, pGL3-SP1-wt) or mutant (5′-T*GTG*CCC-3′, pGL3-SP1-mut) segment of SP-1 mRNA (7386–7596 base, Genbank accession NM_138473.2), which were inserted within the 3′-UTR of the luciferase construct, respectively. The wt or mutant reporter plasmid was co-transfected into normal endothelial cells along with the 27nt miRNA or the control.

**Figure 4:**
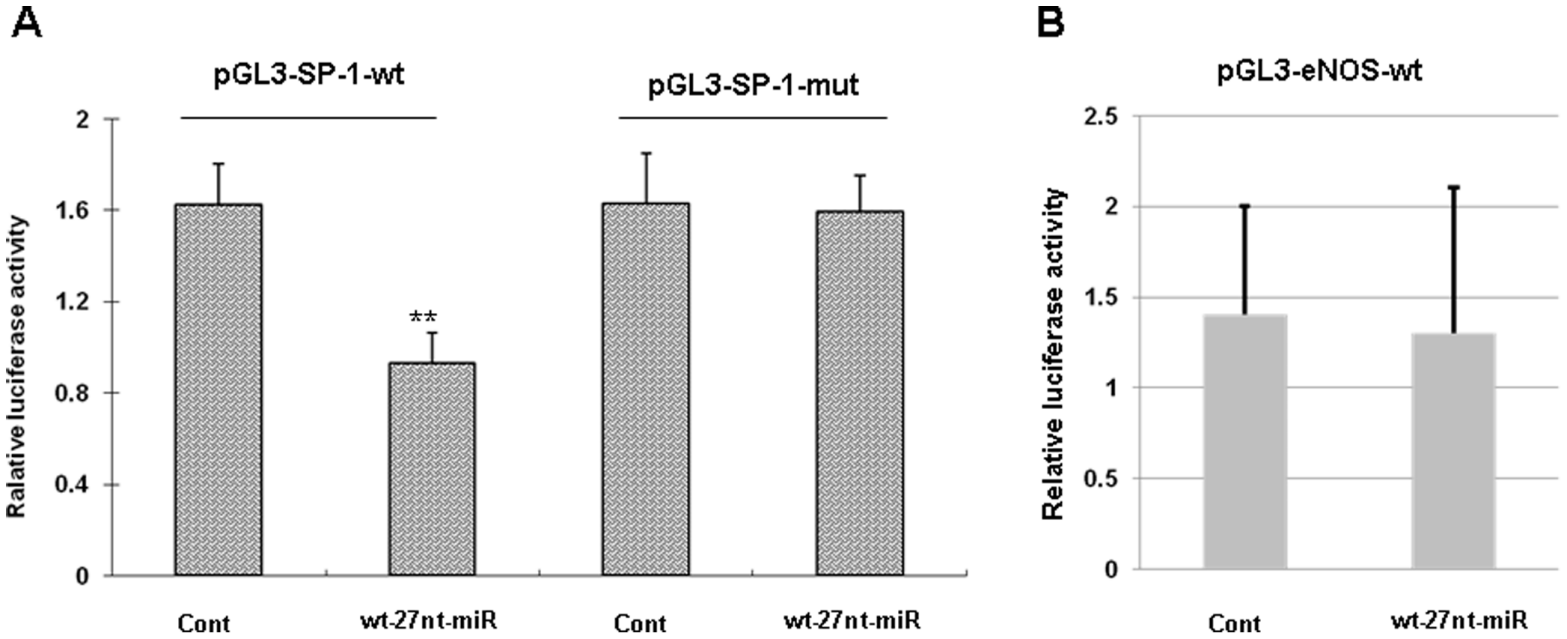
Sp-1 is a direct target of 27-nt-miRNA. (A) Luciferase-reporter plasmids were constructed by inserting the wt (5′-TCACCCC-3′, pGL3-SP1-wt)or mutant (5′-T*GTG*CCC-3′, pGL3-SP1-mut) segment of SP-1 mRNA containing 27nt-miRNA-matched sequence. (B) Luciferase-reporter plasmids were constructed by inserting the eNOS sequence into the vector. The wt or mutant reporter plasmid was co-transfected into normal endothelial cells along with 27 miRNA or negative control. The normalized luciferase activity in the control group was set as relative luciferase activity. All data are representative of three independent experiments. cont, cells transfected with control plasmid; wt-27nt-miR, cells transfected with plasmid expressing wild-ytpe 27-nt-miRNA. **, *P*<0.01 vs control (n = 3).

As shown in Fig. 4A, the 27nt miRNA significantly decreased the activity when co-transfected with the wt reporter plasmid, compared with control (0.93±0.05 vs 1.62±0.07, *P*<0.01). Meanwhile, the SP-1 mutant reporter plasmid abolished 27nt miRNA -mediated decrease in luciferase activity (1.63±0.05 vs 1.59±0.06, *P* = 0.19). These results suggest that 27nt miRNA suppresses SP-1 by directly binding to SP-1 mRNA. Therefore, SP-1 may be a direct target of 27nt miRNA.

To determine if eNOS gene is directly regulated by 27nt miRNA, luciferase-reporter plasmids that contain the sequence of eNOS gene within the 3′-UTR of the luciferase construct were constructed. The eNOS plasmids were transfected into normal endothelial cells along with 27nt miRNA or the control. As shown in Fig. 4B, 27nt miRNA only slightly affected, if any, the activity when compared with control (1.47±0.04 vs 1.38±0.07, *P*>0.05). These results suggest that 27nt miRNA may not direct target the eNOS gene.

### The 27nt miRNA-reduced Endothelial Cell Proliferation and eNOS Expression are Rescued by Sp-1

We have previously reported that 27nt miRNA can inhibit vascular endothelial cell proliferation [16]. Because the SP-1 expression was inhibited by the 27nt miRNA, we wondered if the negative regulation of 27nt miRNA on SP-1 would be related to the endothelial cell proliferation stimulated by vascular endothelial growth factor (VEGF). In this experiment, the cell lines with the 27nt miRNA expression were co-transfected with SP-1 plasmid, then the eNOS mRNA and protein, as well as the cell proliferation were analyzed.

**Figure 5:**
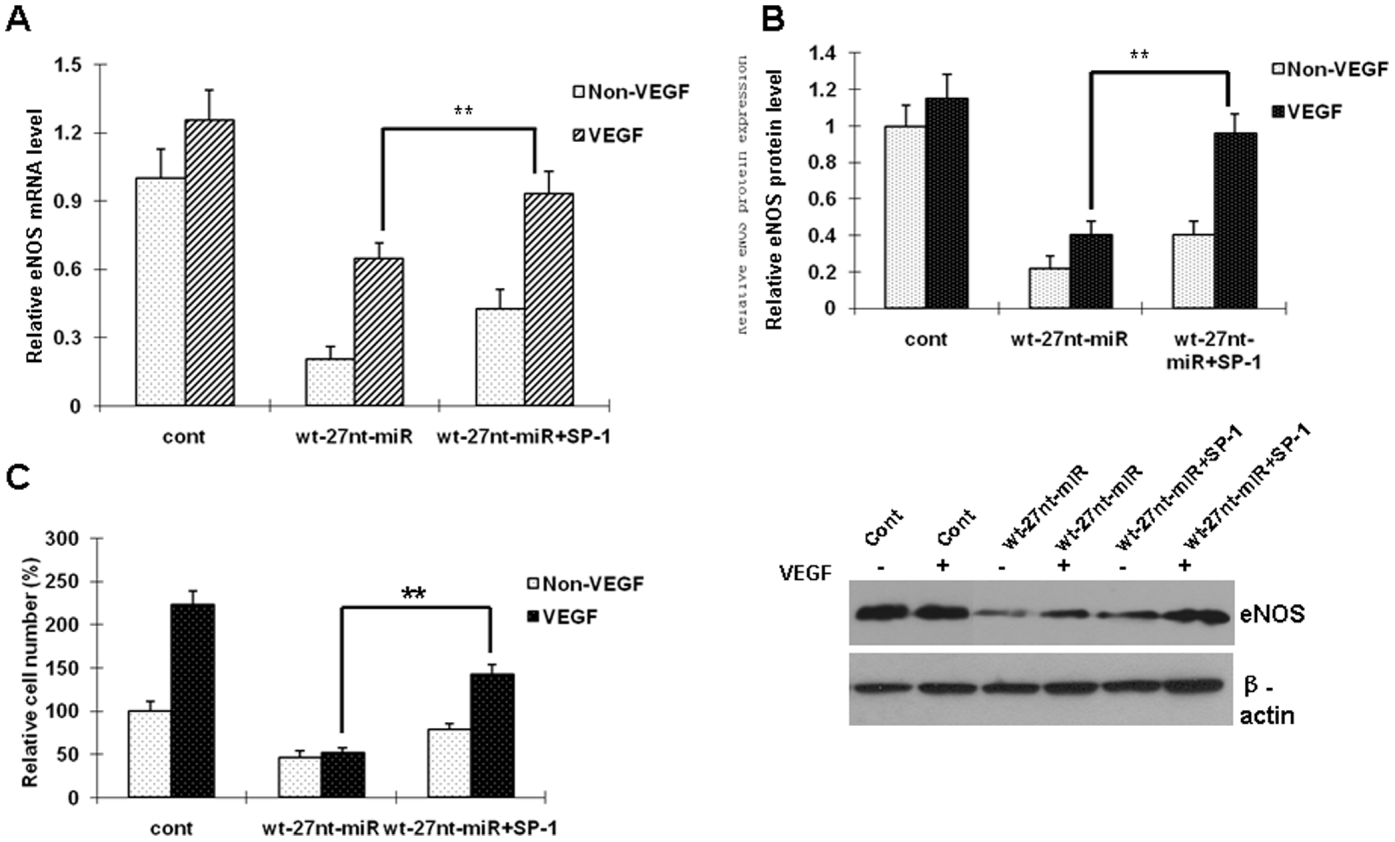
miRNA-reduced endothelial cell proliferation and eNOS expression are rescued by transient SP-1 expression. The cellswere transfected with 27nt miRNA alone or together with the SP-1 plasmid. The eNOS mRNA and protein, as well as the cell proliferation were analyzed. cont, cells transfected with control plasmid; wt-27nt-miR: cells transfected with plasmid of wild-ytpe 27-nt-miRNA; wt-27nt-miR+Sp-1: cells co-transfected with plasmids of wild-ytpe 27-nt-miRNA and SP-1. **, *P*<0.01 vs wt-27nt-miR alone (n = 3).

As shown in Fig. 5A and 5B, under the condition of VEGF stimulation, SP-1 overexpression led to increases in levels of eNOS mRNA and protein by 44.2% (0.932±0.098 vs 0.646±0.067, *P*<0.01) and 136.9% (0.962±0.102 vs 0.406±0.061, *P*<0.01), respectively, when compared with the 27nt miRNA treatment alone.

As expected, the reduced cell proliferation (response to VEGF) by the 27nt miRNA was rescued by SP-1 co-transfection by 178.4% (142.0±12.0 vs 51.0±6.0, *P*<0.01) compared with 27nt miRNA alone condition (Fig. 5C). These results indicate that the decreased SP-1 expression might be responsible for 27nt miRNA-mediated reduction of eNOS expression, and related to the regulation of endothelial cell proliferation by miRNA.

## Discussion

In endothelial cells, the eNOS mRNA levels were decreased by 27-nt miRNA expression, and such inhibitory effects were reversed by treatment with VEGF [14]. The level of 27nt miRNA could be reduced by VEGF or mevastatin in these endothelial cells [14]. Three major findings are described in this study. First, we have shown that 27nt-miRNA derived from the 27nt repeat in intron 4 of eNOS inhibited its host gene expression at the level of transcription. Second, we have shown that nuclear transcription factor SP-1 was a direct target of 27nt-miRNA. There was a possible interaction between intronic miRNAs and SP-1 in the regulation of eNOS expression. Finally, we have shown that 27nt-miRNA could inhibit both eNOS and SP-1 expression, and SP-1 rescues the 27nt-miRNA-induced reduction of vascular endothelial cell proliferation. As we expected, our results suggested that the sequence of 27nt in intron-4 could be a specific critical target for the regulation of eNOS gene expression. To our knowledge, this is the first time it has been reported that an intronic miRNA regulating its host gene expression, which is related to the positive nuclear transcription factor SP-1 in human gene.

The polymorphic miRNA was designed by mutation on the 13, 14, and 15^th^ necleotide that located in the center of the 27-nt-miRNA sequence for two reasons. First, mutations at the 5′- and 3′-terminal regions need to be avoided since these regions are responsible for sequence-specific recognition and functional execution for miRNA-mediated gene silencing, respectively. Second, the 10^th^ nucleotide in mature miRNA is usually the binding site for Argonautes protein, and is at the center of a regular 21-nt miRNA. It was found that the mutant 27nt-miRNA had decreased inhibitory effect on eNOS transcripts *in vitro* as compared with wild-type ones. This phenomenon was also observed in the stable cell lines *in vivo*. These data suggested that the intronic miRNA regulated host gene expression in a sequence-specific manner. Because most TFs function is DNA motif-dependent, and the 27nt-miRNA inhibited eNOS at transcrional level in nuclear, it is reasonable to speculate that TFs might be involved in the 27nt-miRNA-mediated inhibition of eNOS transcription.

TFs and miRNAs are two large families of trans-acting gene regulators in multicellular genomes with extensive interactions on gene regulation [20], [21]. Because most genes in genomes are not controlled by a single trans-acting factor, a model of inter- and intra-combinatorial regulation by TFs and miRNAs has been proposed as a mechanism for regulating gene expression [22]. This dynamic interactive mechanism of gene regulation by TFs and miRNAs has been supported by a few recent observations [18], [19], [23], [24]. In the present study, we focused on SP-1 for two reasons. First, SP-1 has binding sites in the 5′ regulatory regions of eNOS gene, and showed a positive effect on eNOS expression [25]; second, the sequence 5′-TCACCCC-3′at 7443–7449 bases of SP-1 mRNA (Genbank accession NM_138473.2) is matched to the sequence 5′-GGGGTGA-3′ within 27nt miRNA. Since the 27nt-miRNA is derived from eNOS transcripts, we hypothesized that the intronic miRNA and SP-1 shared the transcriptional machinery for eNOS transcription. In other words, it would be reasonable to speculate that the intronic miRNA and Sp-1 interactively regulate eNOS expression.

The common nuclear locations of both TFs and the 27ntintronic miRNA increases the likelihood of their interactions. The 27ntmiRNA has been shown to be able to enter the nucleus by transfection [14] or via reverse transportation [26]. As predicted, our data showed that miRNA overexpression substantially suppressed eNOS expression in association with decreased levels of SP-1. Furthermore, the effect of SP-1 on regulation of eNOS expression was associated with mutation-polymorphisms of miRNA. Using a luciferase activity reporter system, we found that a segment of SP-1 mRNA is the direct target of the 27nt-miRNA. Our data strongly suggest that SP-1 positively, while the 27nt-miRNA negatively, regulates eNOS expression. These effects of the 27nt-miRNA were, at least in part, sequence-specific dependent. In other words, SP-1 and the intronic miRNA might be the two opposite factors to keep balance of eNOS expression regulation under normal physiological condition, i.e, SP-1 is positive, while 27nt miRNA is negative for the eNOS expression. These findings provided a novel mechanistic paradigm on the regulation of host gene expression by intronic miRNA and TFs.

Proliferation of vascular endothelial cells plays important role in a variety of biological or pathological process, including cardiovascular diseases, such as atherosclerosis and restenosis after angioplasty [27], [28]. Multiple targeting genes have been identified during endothelial cell proliferation [29]–[32]. Recently several reports have shown that miRNAs play an important role in regulation of vascular endothelial cell proliferation or apoptosis [33]–[35]. More recently, increasing data showed that miRNAs can affect cell proliferation by targeting TFs in cancer or embryonic stem cells [36]–[40]. Our previous work also showed that the intronic miRNA could regulate endothelial cell proliferation via inhibiting STAT3 signaling pathway [16]. In the current study, we further confirmed the 27nt-miRNA is involved in the regulation of endothelial cell proliferation, while the restoration of SP-1 could rescue the 27nt-miRNA-mediated reduction of endothelial cell proliferation. These results suggest that the intronic miRNA and SP-1 regulate eNOS expression and endothelial cell proliferation. The 4aa genotype (4x 27 repeats) of eNOS was recognized to be a protective for cardiac syndrome X [41]. We therefore proposed that mutation in the 27 bp repeats could be another type of the polymorphism of the eNOS gene, by which the eNOS expression might be increased due to the mutant 27nt miRNA. It has been well known that eNOS expression is a key factor related to cardiovascular diseases. The mutation of 27 bp in the intron 4 will lead to less inhibition of eNOS expression, leading to increased basic release of nitric oxide, consequently being beneficial to promotion of vascular relaxing in condition of hypertension. In summary, we have demonstrated that the 27nt intronic miRNA from intron 4 plays an important role in the regulation of eNOS gene expression. The effect of miRNAs on eNOS expression occurred at both the mRNA and transcription levels, and at least in part through targeting SP-1. The present study provides a novel approach to identifying the potential molecular targets that regulate the expression of disease-associated human genes with polymorphic intron-derived miRNA.

## References

[1] Bartel DP (2004) MicroRNAs: genomics, biogenesis, mechanism, and function. Cell 116 (2): 281–297. 10.1016/s0092-8674(04)00045-5 14744438

[2] Ruvkun G (2008) The perfect storm of tiny RNAs. Nat Med 14 (10): 1041–1045. 10.1038/nm1008-1041 18841145

[3] Ambros V, Lee RC, Lavanway A, Williams PT, Jewell D (2003) MicroRNAs and other tiny endogenous RNAs in C. elegans. Current Biology 13 (10): 807–818. 10.1016/s0960-9822(03)00287-2 12747828

[4] Ying SY, Lin SL (2004) Intron-derived microRNAs-fine tuning of gene functions. Gene 342: 25–28. 15527961 10.1016/j.gene.2004.07.025

[5] Clement JQ, Qian L, Kaplinsky N, Wilkinson MF (1999) The stability and fate of a spliced intron from vertebrate cells. RNA 5 (2): 206–220. 10.1017/s1355838299981190 PMC1369753 10024173

[6] Barik S (2008) An intronic microRNA silences genes that are functionally antagonistic to its host gene. Nucleic Acids Res 36 (16): 5232–5241. 10.1093/nar/gkn513 PMC2532712 18684991

[7] Marsden PA, Heng HH, Scherer SW, Stewart RJ, Hall AV, et al (1993) Structure and chromosomal localization of the human constitutive endothelial nitric oxide synthase gene. J Biol Chem 268 (23): 17478–17488. 7688726

[8] Nakayama M, Yasue H, Yoshimura M, Shimasaki Y, Kugiyama K, et al (1999) T-786–>C mutation in the 5′-flanking region of the endothelial nitric oxide synthase gene is associated with coronary spasm. Circulation 99 (22): 2864–2870. 10.1161/01.cir.99.22.2864 10359729

[9] Kim IJ, Bae J, Lim SW, Cha DH, Cho HJ, et al (2007) Influence of endothelial nitric oxide synthase gene polymorphisms (-786T>C, 4a4b, 894G>T) in Korean patients with coronary artery disease. Thromb Res 119 (5): 579–585. 10.1016/j.thromres.2006.06.005 16842840

[10] Benedetto FA, Testa A, Tripepi G, Mallamaci F, Maas R, et al (2007) An additive effect of endothelial nitric oxide synthase gene polymorphisms contributes to the severity of atherosclerosis in patients on dialysis. Spoto B. Am J Hypertens 20 (7): 758–763. 10.1016/j.amjhyper.2007.02.009 17586410

[11] Rittig K, Holder K, Stock J, Tschritter O, Peter A, et al (2008) Endothelial NO-synthase intron 4 polymorphism is associated with disturbed in vivo nitric oxide production in individuals prone to type II diabetes. Horm Metab Res 40 (1): 13–17. 10.1055/s-2007-1004527 18095216

[12] Wang XL, Sim AS, Badenhop RF, McCredie RM, Wilcken DE (1996) A smoking-dependent risk of coronary artery disease associated with a polymorphism of the endothelial nitric oxide synthase gene. Nat Med 2: 41–45. 8564837 10.1038/nm0196-41

[13] Wattanapitayakul SK, Mihm MJ, Young AP, Bauer JA (2001) Therapeutic implications of human endothelial nitric oxide synthase gene polymorphism. Trends Pharmacol Sci 22: 361–368. 11431031 10.1016/s0165-6147(00)01692-8

[14] Zhang MX, Ou H, Shen YH, Wang J, Wang J, et al (2005) Regulation of endothelial nitric oxide synthase by small RNA. Proc Natl Acad Sci USA 102 (47): 16967–16972. 10.1073/pnas.0503853102 PMC1287968 16284254

[15] Ou H, Shen YH, Utama B, Wang J, Wang X, et al (2005) Effect of nuclear actin on endothelial nitric oxide synthase expression. Arterioscler Thromb Vasc Biol 25 (12): 2509–2514. 10.1161/01.ATV.0000189306.99112.4c PMC1382336 16210567

[16] Yan L, Hao H, Elton TS, Liu Z, Ou H (2011) Intronic microRNA suppresses endothelial nitric oxide synthase expression and endothelial cell proliferation via inhibition of STAT3 signaling. Mol Cell Biochem 357 (1–2): 9–19. 10.1007/s11010-011-0870-x 21611796

[17] Karantzoulis-Fegaras F, Antoniou H, Lai SL, Kulkarni G, D’Abreo C, et al (1999) Characterization of the human endothelial nitric-oxide synthase promoter. J Biol Chem 274: 3076–3093. 9915847 10.1074/jbc.274.5.3076

[18] Aguda BD, Kim Y, Piper-Hunter MG, Friedman A, Marsh CB (2008) MicroRNA regulation of a cancer network: Consequences of the feedback loops involving miR-17–92, E2F, and Myc. Proc Natl Acad Sci USA 105 (50): 19678–19683. 10.1073/pnas.0811166106 PMC2598727 19066217

[19] Taguchi A, Yanagisawa K, Tanaka M, Cao K, Matsuyama Y, et al (2008) Identification of hypoxia-inducible factor-1 alpha as a novel target for miR-17–92 microRNA cluster. Cancer Res 68 (14): 5540–5545. 10.1158/0008-5472.CAN-07-6460 18632605

[20] Hobert O (2008) Gene regulation by transcription factors and microRNAs. Science, 319 (5871): 1785–1786. 10.1126/science.1151651 18369135

[21] Makeyev EV, Maniatis T (2008) Multilevel regulation of gene expression by microRNAs. Science 319 (5871): 1789–1790. 10.1126/science.1152326 PMC3139454 18369137

[22] Zhou Y, Ferguson J, Chang JT, Kluger Y (2007) Inter- and intra-combinatorial regulation by transcription factors and microRNAs. BMC Genomics 8: 396. 17971223 10.1186/1471-2164-8-396 PMC2206040

[23] Romania P, Lulli V, Pelosi E, Biffoni M, Peschle C, et al (2008) MicroRNA 155 modulates megakaryopoiesis at progenitor and precursor level by targeting Ets-1 and Meis1 transcription factors. Br J Haematol 143 (4): 570–580. 10.1111/j.1365-2141.2008.07382.x 18950466

[24] Lee YB, Bantounas I, Lee DY, Phylactou L, Caldwell MA, et al (2009) Twist-1 regulates the miR-199a/214 cluster during development. Nucleic Acids Res 37 (1): 123–128. 10.1093/nar/gkn920 PMC2615617 19029138

[25] Zhang R, Min W, Sessa WC (1995) Functional analysis of the human endothelial nitric oxide synthase promoter Sp1 and GATA factors are necessary for basal transcription in endothelial cells. J Biol Chem 270 (25): 15320–15326. 10.1074/jbc.270.25.15320 7541039

[26] Hwang HW, Wentzel EA, Mendell JT (2007) A hexanucleotide element directs microRNA nuclear import. Science 315 (5808): 97–100. 10.1126/science.1136235 17204650

[27] Bai X, Wang X, Xu Q (2010) Endothelial damage and stem cell repair in atherosclerosis.Vascul Pharmacol. 52(5–6): 224–229. 10.1016/j.vph.2010.02.001 20156585

[28] Inoue T, Croce K, Morooka T, Sakuma M, Node K, et al (2011) Vascular inflammation and repair: implications for re-endothelialization, restenosis, and stent thrombosis. JACC Cardiovasc Interv 4 (10): 1057–1066. 10.1016/j.jcin.2011.05.025 PMC3341937 22017929

[29] Atochin DN, Huang PL (2010) Endothelial nitric oxide synthase transgenic models of endothelial dysfunction. Pflugers Arch. 460 (6): 965–974. 10.1007/s00424-010-0867-4 PMC2975487 20697735

[30] Breuss JM, Uhrin P (2012) VEGF-initiated angiogenesis and the uPA/uPAR system. Cell Adh Migr 17 (6): 6. 10.4161/cam.22243 PMC3547900 23076133

[31] Ohkita M, Tawa M, Kitada K, Matsumura Y (2012) Pathophysiological roles of endothelin receptors in cardiovascular diseases. J Pharmacol Sci 119 (4): 302–13. 10.1254/jphs.12r01cr 22863667

[32] Li Y, Yan CH, Li SH, Han YL (2012) CREG: A Possible Candidate for Both Prevention and Treatment of Proliferative Vascular Disease. Curr Mol Med Jul 23. [Epub ahead of print]. 10.2174/156652412803833526 22834829

[33] Staszel T, Zapała B, Polus A, Sadakierska-Chudy A, Kieć-Wilk B, et al (2011) Role of microRNAs in endothelial cell pathophysiology. Pol Arch Med Wewn 121 (10): 361–366. 21946298

[34] Chen K, Fan W, Wang X, Ke X, Wu G, et al (2012) MicroRNA-101 mediates the suppressive effect of laminar shear stress on mTOR expression in vascular endothelial cells. Biochem Biophys Res Commun. 427 (1): 138–142. 10.1016/j.bbrc.2012.09.026 22989749

[35] Ding Z, Wang X, Khaidakov M, Liu S, Mehta JL (2012) MicroRNA hsa-let-7g targets lectin-like oxidized low-density lipoprotein receptor-1 expression and inhibits apoptosis in human smooth muscle cells. Exp Biol Med (Maywood) 237 (9): 1093–1100. 10.1258/ebm.2012.012082 22956623

[36] Wu Z, Sun H, Zeng W, He J, Mao X (2012) Upregulation of MircoRNA-370 Induces Proliferation in Human Prostate Cancer Cells by Downregulating the Transcription Factor FOXO1. PLos One 7 (9): 45825. 10.1371/journal.pone.0045825 PMC3445500 23029264

[37] Huang Y, Chen HC, Chiang CW, Ye CT, Chen SJ, et al.. (2012) Identification of a two-layer regulatory network of proliferation-related microRNAs in hepatoma cells. Nucleic Acids Res [Epub ahead of print]. 10.1093/nar/gks789 PMC3488236 22923518

[38] Zhang X, Zeng J, Zhou M, Li B, Zhang Y, et al (2012) The tumor suppressive role of miRNA-370 by targeting FoxM1 in acute myeloid leukemia. Mol Cancer 11 (1): 56. 10.1186/1476-4598-11-56 PMC3533721 22900969

[39] Kappelmann M, Kuphal S, Meister G, Vardimon L, Bosserhoff AK (2012) MicroRNA miR-125b controls melanoma progression by direct regulation of c-Jun protein expression. Oncogene [Epub ahead of print]. 10.1038/onc.2012.307 22797068

[40] Chang HM, Martinez NJ, Thornton JE, Hagan JP, Nguyen KD, et al (2012) Trim71 cooperates with microRNAs to repress Cdkn1a expression and promote embryonic stem cell proliferation. Nat Commun 3: 923. 22735451 10.1038/ncomms1909 PMC3518406

[41] Sinici I, Atalar E, Kepez A, Hayran M, Aksoyek S, et al (2010) Intron 4 VNTR polymorphism of eNOS gene is protective for cardiac syndrome X. J Investig Med. 58: 23. 10.2310/JIM.0b013e3181c6197f 19907345

